# Definitions and rates of treatment failure in females with uncomplicated urinary tract infection: a systematic literature review

**DOI:** 10.1093/jac/dkag112

**Published:** 2026-04-08

**Authors:** Meghan E Luck, Amber Martin, Salima Punja, Joanna Kamar, Priccila Zuchinali, Amy G Edgecomb, Jeffrey J Ellis

**Affiliations:** GSK, Collegeville, PA, USA; Evidence Synthesis, Evidera Inc., Waltham, MA, USA; Evidence Synthesis, Evidera Inc., Waltham, MA, USA; Evidence Synthesis, Evidera Inc., Waltham, MA, USA; Evidence Synthesis, Evidera Inc., Waltham, MA, USA; GSK, Collegeville, PA, USA; GSK, Collegeville, PA, USA

## Abstract

**Background:**

Uncomplicated urinary tract infections (UTIs) affect ∼50%–60% of women. Treatment failure can have adverse effects on antibiotic resistance, healthcare utilization and quality of life. The lack of consistently applied definitions of treatment failure prevents comparisons between studies of UTI treatments.

**Objectives:**

We conducted a systematic literature review to investigate definitions of treatment failure in UTI and the corresponding failure rates.

**Methods:**

MEDLINE, Embase and CENTRAL databases were searched from 2011 to 2024 and relevant conference proceedings from 2021 to 2024 for English language studies reporting rates of treatment failure in females aged ≥12 years with uncomplicated UTI.

**Results:**

Publications included reported 14 clinical trials, 11 non-interventional observational studies with chart review and 10 healthcare database studies. Treatment failure definitions were classified as microbiological, clinical, antibiotic prescription based or a composite of these. Evaluation timepoints typically ranged from 1 to 30 days post-treatment. In clinical trials, failure rates varied from 0.8% to 83%, often with marked differences between microbiological, clinical and prescription definitions within the same trial. Rates of treatment failure using combined endpoints, including prescription failure with a healthcare encounter in database studies, were generally more consistent (6.87%–16.7%). Few studies assessed time to symptom resolution. Prescription failure or additional healthcare visits frequently occurred after a median 2–4 weeks.

**Conclusions:**

Treatment failure definitions have been variably defined in the literature. Symptom scores in clinical trials and the need for additional antibiotics or healthcare visits are meaningful outcomes that could underly treatment failure definitions in future studies. The optimal time for each outcome assessment needs further evaluation.

## Introduction

Uncomplicated urinary tract infections (UTIs) were, until recently, usually defined as bladder infections in non-pregnant women without known anatomical or functional abnormalities of the renal tract or co-morbidities.^1^ UTIs affect ∼50%–60% of women during their lifetime, and ∼50% will experience recurrent infections.^2^ In a single year, >11 million women in the USA were estimated to have had at least one UTI requiring antibiotics.^2^ UTIs are the most common infection-related diagnosis in women, a leading cause of antibiotic use, and they incur a substantial disease burden in terms of physician visits, sick days and reduced productivity.^3,4^

Uncomplicated UTIs typically present with symptoms of dysuria, frequency, urgency, haematuria, suprapubic pain and tenesmus. The diagnosis is often made based on symptoms alone or combined with a positive urine dipstick test for the presence of nitrites and/or leucocyte-esterase.^5^ Guidelines differ on the need for confirmatory urine culture when typical symptoms are present.^6^ Management of UTIs is usually based on empirical prescribing of antibiotics.^1^ However, antibiotic treatment failure is a clinical concern and can lead to additional healthcare consultations, and have negative effects on quality of life. Understanding treatment failure rates can facilitate comparisons between studies of therapies used to treat UTI. However, the use of widely differing definitions of both UTI and treatment success in clinical trials currently prevents such comparisons.^7,8^

In 2022, a set of core outcomes for UTI was published under the Core Outcome Measures in Effectiveness Trials (COMET) initiative.^9,10^ Astonishingly, a total of 124 different outcomes across 18 domains were identified. From these, the consensus identified six outcome measures proposed for incorporation into all clinical trials: time (days) from initiation of treatment to resolution of symptoms, recurrence of UTI symptoms following initial resolution in the first 28 days after the start of treatment, worsening or progression of UTI symptoms, self-reported quality of life, treatment satisfaction and adverse events. Guidelines from the FDA and EMA recommend that both symptoms and microbiological criteria be included in primary outcome measures in clinical trials evaluating UTI treatments.^11,12^ Microbiological success is defined by both organizations as a reduction in the causative uropathogen to <10^3^ colony-forming units (cfu)/mL in urine culture. Specific guidance as to the number, type and quantitation of symptoms to define treatment success is lacking.^11,12^ On the other hand, such definitions have little application in real-world practice where urine culture is not always performed and where failure of symptom resolution leading to re-presentation is likely to be the most common indicator of treatment failure.

We conducted a systematic review of the literature to investigate the definitions of treatment failure in UTI and the corresponding rates of failure. We attempted to group definitions thematically and assess the impact on reported failure rates. Our aim was to facilitate the identification of a clinically meaningful definition of treatment failure that could be used in real-world observational studies.

## Methods

### Search methods and study selection

This systematic literature review was conducted according to Preferred Reporting Items for Systematic reviews and Meta-Analyses (PRISMA) guidelines and the *Cochrane Handbook for Systematic Reviews of Interventions*.^13,14^ We searched the Embase, MEDLINE/MEDLINE In-Process and the Cochrane Central Register of Controlled Trials (CENTRAL) databases in OvidSP (http://ovidsp.ovid.com/) for peer-reviewed articles published between 1 January 2011 and 9 January 2024. Searches used a combination of free-text terms and controlled vocabulary terms specific to each database (Supplementary data). Search strings were developed using guideline-recommended filters for specific search platforms to identify evidence from clinical trials and observational studies. Studies were limited to those in humans and published in English.

Supplementary grey literature searches were conducted from January 2021 to January 2024. Separate searches were conducted via OvidSP for relevant conferences indexed in Embase.com (Supplementary data). Conference websites or other relevant media were searched for conferences not indexed in Embase. A single reviewer assessed all identified grey literature, and a second reviewer validated relevant materials. Bibliographies of systemic literature reviews and meta-analyses published in the last 5 years were also reviewed for potential references.

Titles and abstracts were checked against the population, intervention, comparator, outcome and study design criteria (Table 1) to determine eligibility, and the full text of potentially relevant articles was reviewed. Two independent reviewers performed both the title/abstract and full-text phases of study selection, and a third senior investigator made the final judgement in case of disagreement. Study quality was evaluated using the Cochrane Risk of Bias tool for randomized-controlled trials (RCTs), the JBI Critical Appraisal Tool for non-randomized or observational studies without a comparative arm, and the Newcastle-Ottawa Scale for non-randomized or observational studies with a comparative arm.^15–17^

**Table 1.** PICOS eligibility criteria

Criteria	Inclusion criteria	Exclusion criteria	Exclusion reason
Population	Adolescent and adult (≥12 years) females diagnosed with uUTI or acute cystitis, Subgroups of interest include: age >50 years, history of UTI recurrence, MDR, diabetes, drug (antibiotic) allergy	Paediatric populations (<12 years) Pregnant women Patients with febrile UTI Patients with pyelonephritis Indications other than uUTI or acute cystitis Men	Population not of interest
Interventions/comparators	Any, none required	Not applicable	Not applicable
Outcomes	Failure Failure rate with definition used in study Risk factors for treatment failure^a^ Recurrence rate^a^ Risk factors for recurrence^a^ Rate of AMR^a^ Rate of MDR, including definition of MDR^a^ Risk factors for AMR/MDR^a^	Outcomes other than those listed in the inclusion criteria Studies evaluating a mixed population, but results are not reported separately for population of interest	Outcomes not of interest Outcomes not separable for the population of interest
Study design	Observational studies: prospective and retrospective cohorts evaluating at least 20 patients Clinical trials (RCTs or single-arm and non-randomized trials) evaluating at least 10 patients per treatment arm	*In vitro*, *ex vivo*, animal or pharmacokinetic studies, phase I trials, gene studies, case reports/case series Observational studies evaluating <20 patients Clinical trials evaluating <10 patients per arm SLRs	Study design not of interest
Publication type	Articles Conference abstracts^a^	Trial protocols Trial registries Guidelines Narrative reviews Editorials, comments, notes and letters Errata	Publication type not of interest
Language limit	English	Non-English	Non-English language
Date limit	January 2011 to January 2024	Studies published before January 2011	Publication year not of interest

AMR, antimicrobial resistance; MDR, multidrug resistance; PICOS, population, intervention, comparator, outcome and study; RCT, randomized controlled trial; SLRs, systematic literature reviews; (u)UTI, (uncomplicated) urinary tract infection.

^a^Outcomes that were included in the main primary search but which are not included in the present report.

**Figure 1.**
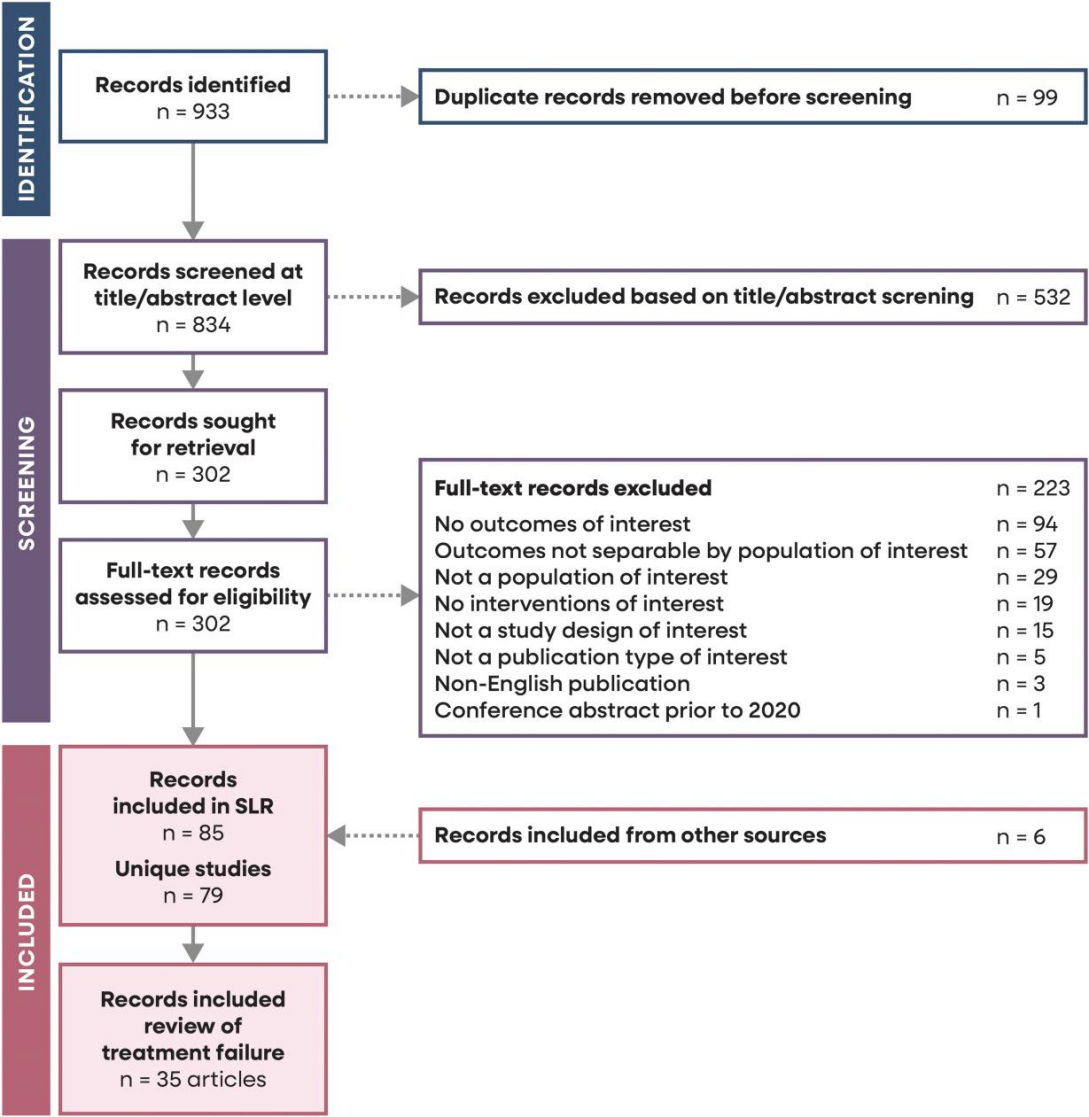
PRISMA flow diagram. SLR, systematic literature review.

The search was conducted as part of a study with wide-ranging objectives encompassing treatment failure, recurrence and antimicrobial resistance in women with uncomplicated UTI. Here, we report a subset of search results pertaining to treatment failure (Figure 1).

### Data extraction

One reviewer extracted the data into a customized data extraction template designed in Microsoft Excel and a second reviewer validated each entry against the source article to confirm accuracy and completeness of data capture. Control measures were used to ensure data quality and consistency. A third reviewer was consulted to resolve any disagreements.

## Results

The primary search identified 834 unique articles of which 35 provided information about uncomplicated UTI treatment failure from 34 studies (Figure 1). Fourteen studies were conducted in North America, ten in Asia, seven in Europe, and three were from other regions or multinational. There were fourteen clinical trials (Table S1, available as Supplementary data at *JAC* Online), and eleven articles reported non-interventional observational studies with chart review (Table S2), mostly conducted in outpatient, primary care, urology clinics and hospitals. Ten further observational studies used real-world healthcare databases as the data source. One additional publication reported risk factors for failure.

Definitions of treatment failure fell into three broad categories: clinical definitions where failure was typically characterized by the failure of symptom resolution, worsening of symptoms, disease progression [for example, progression to pyelonephritis or intravenous (IV) antibiotics] or re-presentation to a healthcare facility (clinical failure); microbiological definitions based on the persistence of pyuria as evidenced by dipstick testing, white blood cell counts on urine microscopy or ≥10^3^ bacterial cfu/mL in urine (microbiologic failure); and treatment-based definitions where failure was defined as an additional antibiotic prescription within a specified period (prescription failure). The timepoints used for the determination of treatment success were variable. Some studies specified both early (usually within 14 days) and late endpoints (30 days) for evaluation of treatment success. For the purposes of this review, we considered failure occurring after 30 days as recurrent infection, and these data were not included.

Since comparative treatment efficacy was not an objective of this review, treatment failure rates were presented as ranges for multi-group studies.

Assessment of quality found that one RCT was at overall high risk of bias,^18,19^ whereas the remainder were of overall low risk or some concerns (Table S3). Among non-randomized or observational studies without a comparator, confounding factors were often not adequately identified or addressed, follow-up was inconsistently reported or incomplete, and strategies for managing incomplete follow-up were generally absent. While exposures and outcomes were typically measured in a valid and reliable way, none of the studies adequately addressed risk of bias across all elements of design, conduct and analysis (Table S4). For non-randomized or observational studies with a comparative arm, most studies were judged to be of good quality, meeting most criteria for selection, comparability and outcome assessment. However, a small number of studies were rated as poor quality due to lack of comparability between cohorts and limited outcome assessment (Table S5).

A summary of the antibiotic regimens tested and reported antimicrobial resistance rates is provided in Table S6.

### Treatment failure in clinical trials

Fourteen trials reported in 13 articles assessed the effectiveness of various antibiotic and non-antibiotic treatments in UTIs. Twelve trials enrolled women from the age of either 18 or 20 years, and one (two studies) enrolled adult and adolescent females from 12 years of age (Table 2). Upper age limits ranged from 40 years to unlimited. Two Phase 3 randomized trials of investigational antibiotics^22,31^ classified patients with missing outcomes as treatment failures, which probably inflated their reported failure rates.

**Table 2.** Treatment failure in clinical trials of uncomplicated UTIs in women

Reference	Age (years)	Evaluation timepoint after treatment completion	*N*	Microbiologic failure		Clinical failure		Prescription failure		Combined endpoint	
				Definition	Failure rate (%)	Definition	Failure rate (%)	Definition	Failure rate (%)	Definition	Failure rate (%)
Abrahamian, 2011^20^	18–40	1–3 days	112	—	—	—	—	—	—	Worsening, persistent, or recurring symptoms and antimicrobial switch	30.9 (S), 54.8 (R)
		3–7 days	98–112	≥10^3^ cfu/mL of UP	(S) 4.1, (R) 40	—	—	—	—		34.6 (S), 54.8 (R)
		4–6 weeks	98–112	≥10^3^ cfu/mL of UP	(S) 26.0, (R) 48.0	—	—	—	—		43.2 (S), 54.8 (R)
Choi, 2015^21^	20–65	2 days	294	—	—	Responded ‘about the same or worse’ at the follow-up visit	15.3	—	—	—	—
Dunne, 2023^22a^	≥18	7–9 days	1071	≥10^3^ cfu/mL of the baseline UP	9.7, 14.3 (S) 3.9, 12.7 (R) 18.4, 27.3	Ongoing symptoms	9.9, 10.6 (S) 10.1, 10.3 (R) 9.4, 11.6	Received non-study antibacterials	0.8, 2.9 (S) 1.1, 1.2 (R) 0, 7.9	Clinical and microbiologic	4.4, 5.2 (S) 1.0, 4.9 (R) 3.4, 18.0
										Clinical, microbiologic, prescription and indeterminate outcome	26.9, 29.8 (S) 15.7, 28.4 (R) 33.3, 60.4
Hamasuna, 2014^23^	≥20	5–9 days	154	≥10^3^ cfu/mL	16.6, 28.8	Ongoing symptoms	6.2, 16.4	—	—	—	—
		4–6 weeks in those with cure	122		9.6, 10.0		1.4, 5.8	—	—	—	—
Hassan, 2022^18^	20–70	28 days	46	—		Ongoing symptoms	34.78	—	—	—	—
Hooton, 2012^24^	18–55	5 days	300	≥10^5^ cfu/mL of all UPs and <10-fold decrease in the causative UP	4, 19	—	—	Required antimicrobial treatment during follow-up	7, 12	—	—
		30 days	300	—	—	—	—		7, 18	—	—
Huttner, 2018^25^	≥18	14 days	494	≥10^3^ cfu/mL	18, 27	—	—	Required additional antibiotic or discontinuation due to lack of efficacy	23, 30	—	—
		28 days	485		26, 37	—	—		27, 39	—	—
Liu, 2019^26^	18–75	1 day–3 weeks	122	Bacterial persistence	11.7, 23.0	<30% of symptoms cured on a 5-point scale	3.3, 4.9	—	—	<70% symptom score reduction or UP present	9.8–18.0
Matsumoto, 2011^27^	≥20	5–9 days	39	Not defined	5.1	Not defined	5.1	—	—	—	—
Monsen, 2014^28^	≥18	1–7 days	1117	Persistence of original species	43, 72	Symptom score of >0	43, 72 (S) 83, (R) 79	—	—	—	—
		28–46 days	805		32, 46		32, 46 (S) 78, (R) 75		—	—	—
Rădulescu, 2020^29^	18–60	1 day	93	—	—	Ongoing symptoms	11.83	—	—	—	—
		21 days	93	—	—		13.33, 6.25 (R) 11.2, 62.5	—	—	—	—
Sadahira, 2016^30^	≥20	5–9 days	104	≥10^3^ cfu/mL	7.5, 13.7	Ongoing symptoms	5.7, 7.8	—	—	—	—
Wagenlehner, 2024^31a^	≥12	6–11 days	1148	≥10^3^ cfu/mL of baseline UP	27.5–42.8	Symptom score of >0	32.1–36.7	Antibiotic switch	1.6–6.5	Clinical and microbiological or additional antimicrobials	41.5–56.4
		21-29 days	1201		47.3–56.7		42.5–45.6		3.6–11.3		56.8–68.5

cfu, colony-forming units; N, number of patients; R, resistant to the prescribed antibiotic; S, susceptible to the prescribed antibiotic; UP, uropathogen; UTI, urinary tract infection.

^a^Patients with missing outcomes were classified as treatment failures.

#### Definitions of treatment failure in clinical trials

Nine trials reported microbiologic failure.^20,22–26,28,30,31^ The most common definition (six trials) was persistence of ≥10^3^ cfu/mL of a uropathogen (either causative or any) at the evaluation timepoint.^20,22,23,25,30,31^ One trial defined microbiologic failure as 10^5^ cfu/mL of all uropathogens, with a <10-fold decrease in the causative uropathogen,^24^ one as persistence of the original pathogen,^28^ and two did not provide a definition.^26,27^

Ten of 13 trials reported clinical failure that was based on failure of symptom resolution by the evaluation date.^18,21–23,26–31^ Three trials defined clinical failure using symptom severity scores; two used 3-point scales with failure defined as a score of >0,^28,31^ and one used a 5-point scale with failure defined as a <30% decrease.^26^

Four trials reported prescription failure during the follow-up period (either an extension of the original antibiotic or a new antibiotic),^22,24,25,31^ and four used composite endpoints of treatment failure that incorporated combinations of clinical, microbiologic and/or prescription endpoints.^20,22,26,31^ Initial evaluation timepoints ranged from 1 to 14 days after completion of the treatment regimen in most (Table 2).

#### Rates of treatment failure in clinical trials

Rates of treatment failure varied both between individual trials, and within trials according to the definition applied. Six trials reported both microbiologic and clinical failure with varying degrees of concordance. For three trials,^22,27,31^ microbiologic and clinical failure rates were within similar ranges (9.7%–14.3% microbiologic failure and 9.9%–10.6% clinical failure in one trial,^22^ 5.1% and 5.1%, respectively, in the second,^27^ and 27.5%–42.8% and 32.1%–36.7% in the third trial^31^). In the other three trials,^23,26,30^ rates of microbiologic failure were observed to be higher than rates of clinical failure (16.6%–28.8% microbiologic failure and 6.2%–16.4% clinical failure in one trial,^23^ 11.7%–23.0% and 3.3%–4.9%, respectively,^26^ and 7.5%–13.7% and 5.7%–7.8% respectively,^30^ in the other two trials).

For most trials, clinical success was defined as symptom resolution without further quantification. Two trials using symptom scores gave widely disparate rates of treatment failure reflecting different methods of scoring ‘improvement’. The percentage of patients with a <30% improvement in their score ranged from 3.3% to 4.9%,^26^ whereas the percentage with a score of 0 (no symptoms) in the second trial was from 32.1% to 36.7%.^31^ An exploratory analysis defining clinical success as a symptom score of ≤1 instead of 0 in this trial decreased the clinical failure rate to 21.7%–24.7%.^31^

Among the four trials reporting prescription failure, the reported rates were aligned with the rate of microbiologic failure in two trials (23%–30% for prescription failure and 18%–27% for microbiologic failure in one trial, and 7%–12% versus 4%–19%, respectively, in the other).^24,25^ By contrast, the two other trials showed markedly disparate results, with the rate of prescription failure from 0.8% to 2.9% versus a microbiologic failure rate of 9.7%–14.3% in one, and 1.6%–6.5% versus 27.5%–42.8%, respectively, in the other.^22,31^

In trials with more than one evaluation timepoint, microbiologic failure rates were generally higher with increasing time since treatment completion. Among four trials with consecutive evaluation of clinical failure, failure rates decreased with increasing time from treatment completion in three^23,28,29^ and increased over time in one trial.^31^

Treatment failure rates using composite endpoints tended to be higher than the individual components when the endpoint included the conjunction ‘or’, and lower when it included ‘and’. Four trials reported that failure rates were generally higher when the causative uropathogen was treatment-resistant.^20,22,28,29^

Few trials reported COMET outcomes. Three trials reported time to symptom resolution. In one trial that assessed efficacy of a 3-day or 5-day treatment regimen, the median time to resolution of all symptoms was similar in both groups at ∼5 days, and occurred in >80% of patients by day 12 (patients received either a 3-day or a 5-day treatment regimen).^22^ One trial reported a median time to symptom improvement (not resolution) of 2.4 days in patients with treatment success.^21^ In the third trial, the duration of symptoms was 2–5 days in approximately three-quarters of participants who were cured, and between 6 and 10 days in the remainder.^18^

### Treatment failure in observational studies

#### Database studies

Ten studies used healthcare databases for source data, of which six were conducted in the USA (Table S2).^32–41^ Eight studies defined UTI as patients with a relevant diagnostic code, and seven also required evidence of an antibiotic prescription.^32–35,37,38,40,41^ One study required a positive urine culture with an antibiotic prescription,^36^ and one study did not specify the inclusion criteria.^39^

Participants were aged from 12 years in six studies and from 18 years in the remainder (Table 3). No study reported microbiologic failure. Four studies reported clinical failure based on the occurrence of severe or complicated disease as indicated by hospital admission, emergency department or emergency clinic visit, or evidence of pyelonephritis.^32,36,38,41^ All studies included an additional antibiotic prescription within a specified timepoint as evidence of treatment failure. Additional antibiotic prescriptions were used alone or in composite endpoints combined with clinical criteria such as use of IV antibiotics, and/or additional visits for UTI including hospitalization and emergency visits. The assessment timepoint in database studies was most frequently 28 days after the index (UTI diagnosis or prescription date), although some studies also evaluated 7- and 14-day timepoints.

**Table 3.** Treatment failure definitions and rates of failure in studies using databases as the data source

Author, year	Age (years)	Assessment timepoint	*N* evaluated	Definition of treatment failure (weighted cumulative risks)					
				Clinical failure	Failure rate (%)	Prescription based	Failure rate (%)	Combined endpoint	Failure rate (%)
Butler, 2021^32^	18–44	30 days	1 140 602	Developed pyelonephritis	0.3–0.5	Required antibiotic switch	8.3–16.3	—	—
Franklin, 2023^33^	≥18	28 days	238 335	—	—	—	—	Additional antibiotic prescription, IV antibiotics, UTI diagnosis in ED or inpatient	12.3
Fromer, 2023^34^	≥12	28 days	376 004	—	—	—	—	Antibiotics, IV antibiotics, UTI diagnosis in ED or inpatient	16.7
Kon, 2022^35^	≥18	28 days	623	—	—	Different antibiotic prescription, excluding extension of index treatment or change due to urine culture result	0–3.85	—	—
Patel, 2023^36^	≥12	28 days after first visit	2880	Hospitalization	8.0	Antibiotic prescription	23.3	—	—
				ED or outpatient clinic visit	23.6				
Ten Doesschate, 2019^40^	≥12	14 days	46 855	—	—	—	—	Second antibiotic prescription + UTI code	5.8–5.9
		28 days							9.6–9.7
Ten Doesschate, 2020^41^	≥12	28 days	21 891	Pyelonephritis code + antibiotic prescription	4.0	—	—	Second antibiotic prescription +/− cystitis or pyelonephritis code	16.3
Shafrir, 2023^38^	≥18	7 days	33 759	Hospital admission	0.5–0.98	Antibiotic switch	3.79–5.59	Hospitalization or ED visit, IV antibiotics, or antibiotic switch	6.87–8.16
				ED	0.89–1.14			Hospitalization or ED visit, IV antibiotics (excluding antibiotic switch)	2.9–3.5
				IV antibiotics	0.08–0.14	—	—	—	—
				Emergency clinic	0.92–1.38	—	—	—	—
				Pyelonephritis	0.27–0.76	—	—	—	—
		8–30 days	33759	—	—	—	—	Reinfection (UTI diagnosis, inpatient, ED or emergency clinic visit)	7.76–9.21
		7–30 days	33759	—	—	—	—	Combined failure	15.9–16.1
Schultze, 2023^37^	≥12	28 days	124 971	—	—	—	—	Antibiotic switch, IV antibiotics, UTI diagnosis in ED or inpatient, UTI progression e.g. pyelonephritis	15.1
Wang, 2020^39^	≥12	28 days	557 669	—	—	—	—	IV antibiotics or different antibiotic prescription, or primary UTI diagnosis within 28 days	14.0

ED, emergency department; IV, intravenous; N, number of patients; UTI: urinary tract infection.

Rates of clinical failure were low in most studies, ranging from 0.27% to 4%. The exception was a US study limited to patients with confirmed extended-spectrum beta lactamase-positive infections, in whom 8% were hospitalized and 23.6% had an additional clinical visit.^36^ Use of IV antibiotics was infrequent (0.08%–0.14%) in one study where this was measured as an individual endpoint,^38^ and rates of prescription failure ranged from 0% to 23.3% (Table 3). The treatment failure rate at day 28 based on composite endpoints (additional antibiotic plus IV antibiotics, medical visit/hospitalization or pyelonephritis code) was remarkably consistent, ranging between 9.6% and 16.7%.

#### Studies using individual-level data (chart review) from electronic healthcare records

Eleven studies used individual patient information extracted from health records (i.e. chart review) from primary care clinics, urology clinics and hospitals, an online telehealth service and a pharmacy registry (Table 4).^42–52^ Nine of the studies were retrospective in design.^42,44–46,48,49,51,52^ Three studies used diagnostic codes for initial patient identification followed by individual-level data extraction.^43,47,50^

**Table 4.** Treatment failure definitions and rates of failure in non-interventional observational studies using individual patient data extracted from health records

Author, year	Age (years)	Evaluation timepoint	*N* evaluated	Definition of treatment failure							
				Microbiologic failure	Failure rate (%)	Clinical failure	Failure rate (%)	Prescription based	Failure rate (%)	Combined endpoint	Failure rate (%)
Ahn, 2021^52^	≥18	7–14 days post Rx	47	Non-sterile culture	17	Ongoing symptoms	21.3	—	—	—	—
Beahm, 2018^42^	≥19	7–11 days post Rx	610	—	—	Ongoing symptoms	11.1	—	—	—	—
Cowart, 2019^43^	18–65	14 days/30 days after Rx initiation	436	—	—	—	—	—	—	Second antibiotic within 14 days or medical visit for UTI within 30 days	10
Daumeyer, 2023^44a^	≥18	7 days after Rx initiation	2193	—	—	Ongoing symptoms	9.2	—	—	—	—
		30 days after Rx initiation		—	—	Returned to the UTI telehealth programme	2.2	—	—	—	—
Etani, 2017^45^	16–<90	Not defined	165	—	—	—	—	—	—	Ongoing symptoms of bladder irritation, bacteriuria, pyuria (≥4 WBCs per hpf)	0.7
Koguchi, 202^46^	≥16	5–7 days post-treatment	223	—	—	—	—	—	—	Ongoing symptoms of bladder irritation, bacteriuria, pyuria (≥4 WBCs per hpf)	6.0
Koh, 2023^47^	18–50	28 days after first visit	3194	—	—	—	—	—	—	Re-attendance with antibiotic prescription or hospitalization for UTI complications	4.57
Kusin, 2023^48b^	18–85	7 days post Rx	64	—	—	UTISA score >2	16–44	Antibiotic prescription	11	—	—
		14 days post Rx	60	—	—		17–31	—	—	—	—
Naber, 2023^49^	≥ 12	Within 28 days after first visit	386	—	—	—	—	Antibiotic prescription	8.8	—	—
Yetsko, 2023^50^	≥18	During treatment (5–7 days)	261	—	—	—	—	—	—	Continued symptoms or new antibiotic	2.3–5.7
		30 days post Rx		—	—	—	—	—	—	Treatment of recurrent symptomatic UTI	10.4–11.3
		Above combined		—	—	—	—	—	—	Above combined	12.7–17
Welch, 2021^51^	≥ 18	14 days post Rx	278	—	—	—	—	—	—	Premature treatment discontinuation, other antibiotics and ongoing signs or symptoms	11.1

hpf, high-powered field; Rx, treatment; UTI, urinary tract infection; UTISA, Urinary Tract Infection Symptom Assessment; WBC, white blood cells.

^a^Included patients with complicated and uncomplicated UTI.

^b^74% of patients had recurrent UTI.

One study reported microbiologic failure defined as ‘non-sterile culture’ with a failure rate of 17%.^52^ Clinical failure was reported in four studies, defined as ongoing symptoms (three studies) or an Urinary Tract Infection Symptom Assessment (UTISA) score ≥2 (one study).^42,44,48,52^ One study additionally used re-presentation as indicative of clinical failure.^44^ Two studies reported prescription failure^48,49^ and six reported composite endpoints that included variable combinations of ongoing symptoms (four studies^45,46,50,51^), an additional antibiotic prescription (four studies^43,47,50,51^), evidence of bacteriuria and pyuria (two studies^45,46^), additional healthcare visits (two studies^43,47^) or premature treatment discontinuation (one study^51^). The evaluation timepoints were within 7–14 days after treatment completion in most studies.

Clinical failure 7–14 days after treatment completion ranged from 9.2% to 21.3% for failure of symptom resolution, and 16%–44% when using the UTISA scoring method. Prescription failure was reported for 8.8%–11% of patients. Composite endpoint failure rates 7–14 days after treatment completion ranged from 2.3% to 11.1%.

Two studies reported time to treatment failure. Median (interquartile range) time to treatment failure in one study (defined as continued symptoms or new antibiotic within 30 days) was 13.5 days (8–17) and 10 days (5–16) for different antibiotic regimens.^50^ In the second study, mean (95% confidence interval) time to treatment failure (defined as re-attendance to primary care or emergency department for urinary symptoms with a same-day antibiotic prescription, or hospitalization for complications of UTI within 28 days) was 27.3 days (27.1–27.5) and 26.8 days (26.5–27.1) for different antibiotic regimens.^47^

### Risk factors for treatment failure

Our search identified one study that specifically evaluated risk factors for treatment failure.^19^ This was a nested case-control study conducted as part of a randomized-controlled antibiotic trial reported above.^19,25^ Diabetes mellitus, a strongly positive urine dipstick result and geographic location (recruited at the Tel Aviv site compared with Geneva or Lodz) all increased the risk of microbiologic failure. However, age ≥52 years was the only independent risk factor identified for clinical and microbiologic failure.^19^

One additional study observed that a small percentage of treatment failures could be attributed to poor treatment adherence by patients.^42^

## Discussion

There was little consistency in the definitions of treatment failure in the studies assessed in this review, making comparisons between studies a challenge given the heterogeneity in definitions. The exception was the conduct of studies using large healthcare databases in which clinical information, such as symptoms, urine dipstick test results and culture results, is not usually captured. All of the database studies we reviewed included prescription failure as an indicator of treatment failure, with six also specifying a proxy for clinical failure, such as a healthcare visit or a second infection code. The treatment failure rates in these studies were relatively consistent across countries and study years.

Small differences in definition appeared to have marked impacts on failure rates. Kon *et al*.^35^ defined treatment failure as a different antibiotic prescription but excluded extensions of the index treatment or change due to a urine culture result, which lead to very low rates of failure (0%–3.85% depending on the initial antibiotic). However, this study used a database holding electronic health record data, and clinical information such as culture results are not typically available in claims-based databases, limiting the applicability of this definition.

There is no available validated symptom scoring tool for UTI. In clinical trials, analysis of different symptom score cut-offs in one study showed that the requirement for complete symptom resolution versus symptom improvement increased failure rates considerably. Most studies did not attempt to score symptom severity, and it is unclear whether complete symptom resolution was required for treatment success. Only half of clinical trials reported prescription failure, but with rates that varied between 0.8% and 30% in the first 14 days after completion of the initial treatment. It is unclear whether these considerable differences reflect different clinical thresholds for additional prescriptions, different antimicrobial susceptibility patterns in the studied population, or different treatment efficacies. Other unknown factors in the study designs or populations under investigation may also have contributed to the observed variability.

Overall, treatment failure rates tended to be higher in the context of a clinical trial than in studies based on real-world data. Most clinical trials included a microbiological failure definition, whereas this was rare (only one study) in real-world studies. Rates of prescription failure showed more alignment between the clinical trial and real-world settings for most studies.

From the few studies that reported the speed of symptom resolution and time to treatment failure, it seems apparent that while symptom improvement may occur early after treatment initiation, complete symptom resolution occurs later.^21,22^ The median time to treatment failure (including prescription failure and additional healthcare visits) ranged between 10 and 27 days in two observational studies that used medical record review, but 4 days in a database study.^47,50,53^ Treatment failure rates are likely to be higher if the evaluation time point is scheduled early after treatment completion, and if the clinical endpoint is complete symptom resolution. Conversely, an evaluation period limited to <14 days is likely to miss a sizeable number of prescription failures. Consistent with this hypothesis, in three out of four clinical trials with early and late evaluation timepoints, clinical failure rates decreased over time.^23,28,29^ The observation that conversely, microbiologic failure rates increased over time after treatment completion raises questions about the clinical validity of follow-up urine culture results in treatment failure definitions in the absence of clinical symptoms.

After we conducted our review, Fromer *et al*.^53^ published results from a US electronic healthcare record database used to investigate risk factors for treatment failure in UTI. Treatment failure was defined as having a new/repeat antibiotic prescription, IV antibiotics of another acute UTI diagnosis ≤28 days following the initial prescription. The risk of treatment failure was significantly higher in patients who had ≥3 previous antibiotic prescriptions (60% increased risk), were treated with fosfomycin (60% increased risk), were diagnosed at the emergency department (49% increased risk), if they lived in southern states (37% increased risk), had a recurrent UTI (12% increased risk) and were obese (6% increased risk). In addition, the risk of treatment failure increased with age. Compared with a reference group aged 12–17 years, treatment failure increased by 9% in 35–49-year-olds, 19% in 50–60-year-olds, 27% in 65–74-year-olds and 35% in women aged 75 years or older. The association between treatment failure and age was also noted in a European/Israeli study in which age ≥52 years was associated with a 2.5–3-fold increase in clinical or microbiologic treatment failure.^19^

Despite the guidelines published by COMET, few studies incorporated these endpoints.^9,10^ The COMET guidelines were formulated based on consensus and are not evidence-based and are not aligned with FDA and EMA guidance for clinical trial outcomes for UTIs. Their implementation could promote better comparability of results between studies, but as yet their uptake has been minimal. We searched clinicaltrials.gov for planned or ongoing studies of uncomplicated UTIs and identified seven studies, of which six were in adult women and three assessed the outcome of UTIs. The primary outcome measure was different in each study; one required resolution of symptoms within a 3-day period, one required a 50% or more improvement in symptom score at 7 days, and the other required resolution of clinical symptoms and microbiologic response at day 10. Therefore, in this small sample of contemporary ongoing studies, there is no evidence of a shift towards harmonization of endpoints.

Our results underscore the need for harmonization of endpoints and definitions used in studies evaluating treatments for UTI, but highlight the challenges in achieving this, particularly for clinical trials and observational studies using patient-level data. While microbiologic failure is an important trial endpoint, its utility in defining treatment failure deserves re-consideration given that there appeared to be inconsistent relationships between microbiologic failure and prescription failure in this review. Furthermore, placebo-controlled trials conducted in the past showed substantial rates of spontaneous bacterial eradication (>28% after 7 days), and unpredictable associations between some symptoms and bacteriuria.^54^ It is accepted that no microbiological test can distinguish between asymptomatic bacteriuria or symptomatic UTI, and urine test results cannot be used in isolation to define UTI or treatment failure.^55^

Quantification of symptom severity using tools such as the UTISA and identification of a clinically meaningful reduction (as opposed to complete resolution) in symptoms would promote better understanding of the impact of treatment on patients.^21^ Compared with the subjective assessment of symptoms, the requirement for an additional prescription or additional healthcare visit appear to be well defined, objective endpoints indicating treatment failure.

Limitations of the data include a paucity of information about risk factors for treatment failure, and the kinetics of cure versus microbiologic, clinical and prescription failure. Many observational studies were limited by confounding and incomplete follow-up, and the potential risk of bias was not addressed. Limitations of our review include the English language restriction and a search period that did not start until 2011. Finally, after our review was conducted, the Infectious Diseases Society of America updated the definition of uncomplicated UTI to ‘Infection confined to the bladder in afebrile women or men.’^56^ Future studies of UTI will need to take this change into account when comparing results with past studies using the previous definition restricted to non-pregnant women.

In conclusion, guidelines for defining treatment success and failure in clinical trials are not necessarily clinically meaningful given their reliance on demonstrating microbiologic cure. Objective endpoints, such as additional antibiotic prescriptions and the need for additional healthcare visits could provide a more clinically meaningful picture of treatment failure. We advocate the use of symptom scoring tools and careful differentiation between definitions of complete symptom resolution versus symptomatic improvement in clinical trials. For real-world studies, the requirement for a new antibiotic prescription and medical visit provides a clinically meaningful endpoint that could be made more specific (for example, excluding extensions of the initial antibiotic) according to the characteristics of the database. Different endpoints indicating treatment success or failure need to occur at different times, with symptomatic improvement expected early, complete symptom resolution expected later, and prescription failure or additional healthcare visits occurring after a median of 2–4 weeks. UTIs affect most women at some point in their lives and are major causes of antibiotic consumption. Harmonization of a clinically relevant definition of treatment failure could help inform comparisons between treatments and inform clinical decision-making.

## Supplementary Material

dkag112_Supplementary_Data

## Data Availability

Data sharing is not applicable to this article, as no datasets were generated or analysed during the current study.

## Supplementary data

Tables S1–S6 are available as Supplementary data at *JAC* Online.
