# Supplementary material for: Definitions and rates of treatment failure in females with uncomplicated urinary tract infection: a systematic literature review
Source: J Antimicrob Chemother. 2026 Apr 8;81(5):dkag112. doi: 10.1093/jac/dkag112 (PMC13069487; doi:10.1093/jac/dkag112)
Supplement: dkag112_Supplementary_Data [file dkag112_supplementary_data.docx]

**SUPPLEMENT**

**Electronic database search strategies**

Embase Search Strategy (via OvidSP)


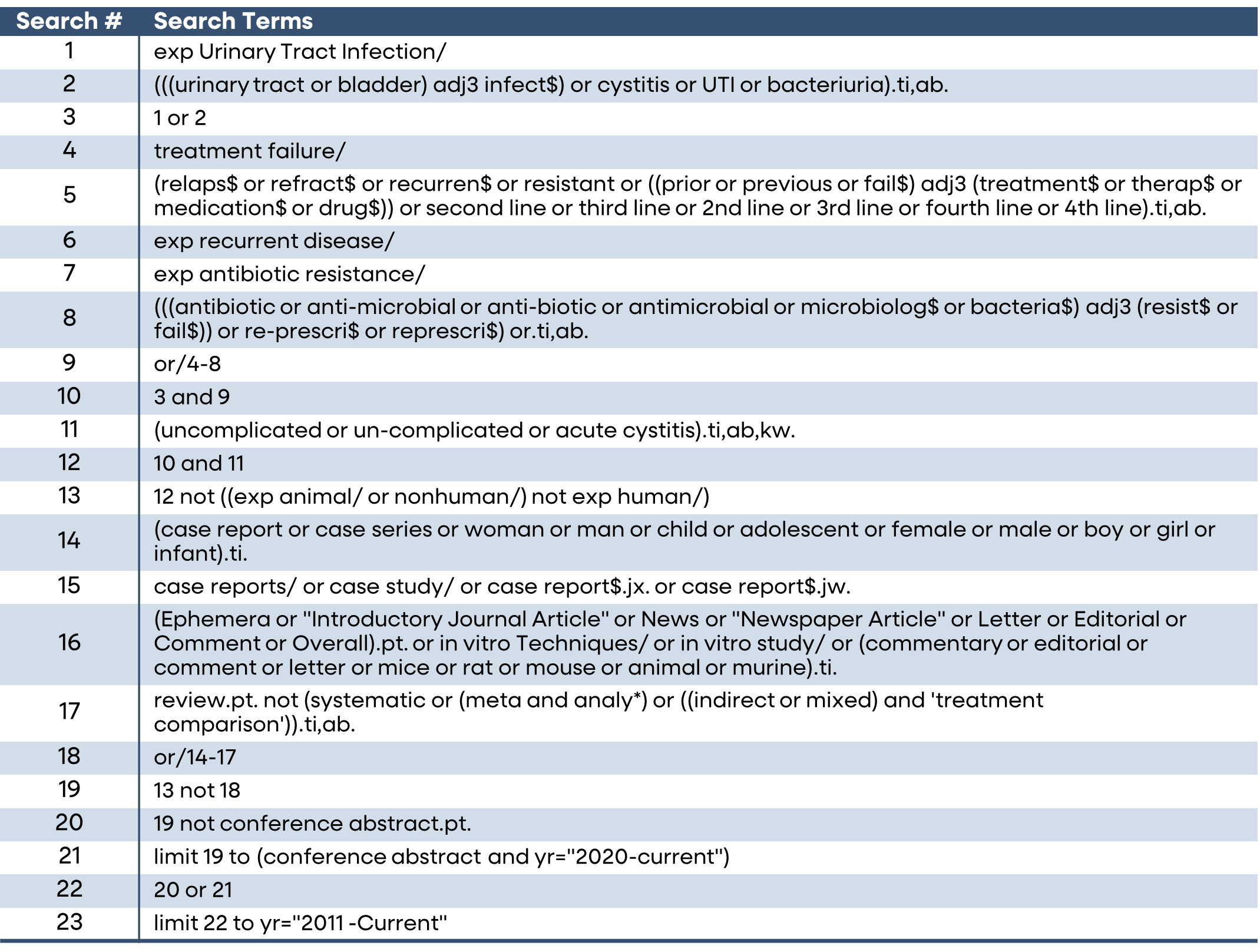


MEDLINE Search Strategy (via OvidSP)


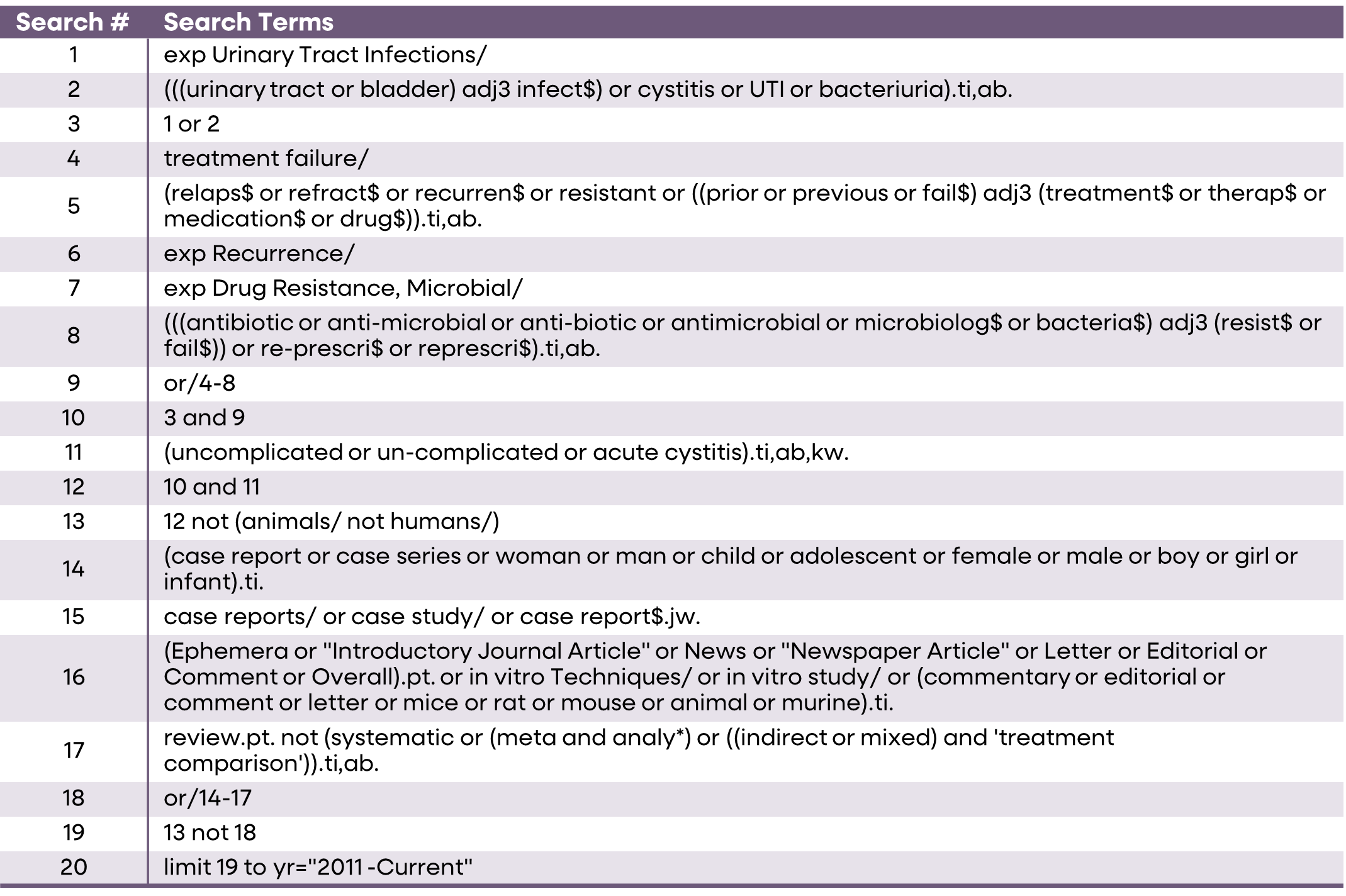


CENTRAL Register of Controlled Trials (via OvidSP)


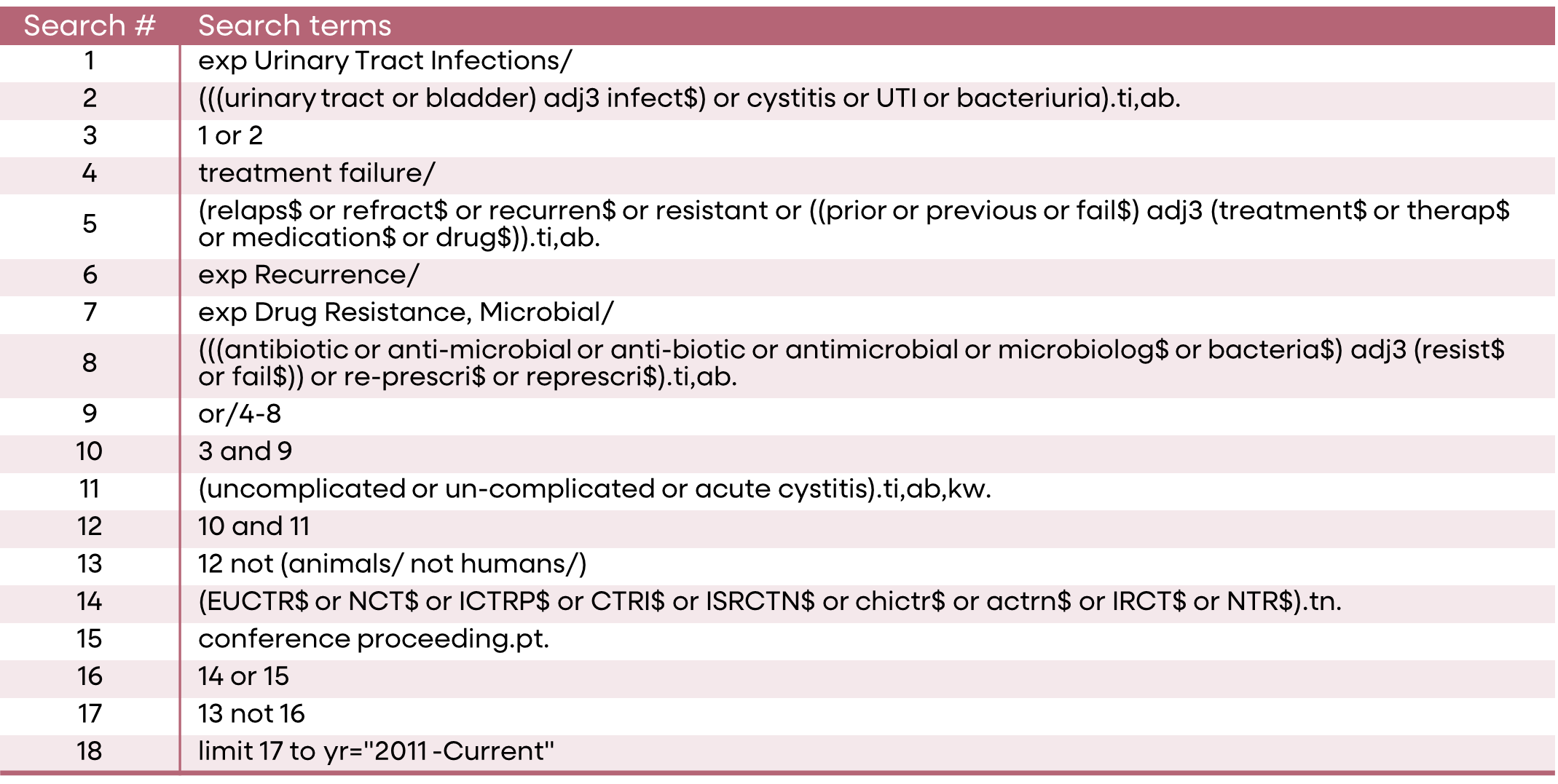


Proceedings from January 2021 to January 2024 were searched for the following conferences:

- IDWeek
- European Society of Clinical Microbiology and Infectious Diseases Annual Meeting
- American Urological Association Annual Meeting
- American Society of Microbiology Microbe Conference
- International Society for Pharmacoeconomics and Outcomes Research Annual Meeting

**Table S1** Summary of key features of clinical trials reporting treatment failure

| **Reference** | **Country** | **Setting** | **Study years** | **N evaluated** | **UTI definition** | | |
| --- | --- | --- | --- | --- | --- | --- | --- |
|  |  |  |  |  | **Microbiology** | **Symptoms** |  |
| Abrahamian, 2011^1^ | US | Emergency department and neighboring clinics | 2002–2005 | 139 | - | Dysuria, frequency, urgency for ≤7 days |  |
| Choi, 2015^2^ | South Korea | 14 centers -not specified | 2010 | 294 | - | Symptoms of acute uncomplicated urinary tract infection for ≤7 days |  |
| Dunne, 2023^3^ | US | 142 centers in 4 countries - not specified | 2018–2020 | 1071 | Urinalysis positive for nitrite and either a positive leukocyte esterase or microscopic evidence of WBCs | ≥2 signs or symptoms |  |
| Hamasuna, 2014^4^ | Japan | 35 centers – not specified | 2010–2011 | 200 | Pyuria (uncentrifuged >10 WBCs/mm^3^, centrifuged >5 WBC/mm^3^) or positive leucocyte esterase result using urine test paper. Bacteria >10^4^ cfu/mL. | Any symptoms |  |
| Hassan, 2022^5^ | Pakistan | Hospital medicine department | 2019–2020 | 46 | - | Presenting with symptoms |  |
| Hooton, 2012^6^ | US | Student health center and a referral center | 2005–2009 | 300 | Pyuria (WBC ≥8 cells/mm^3^) | Any symptoms |  |
| Huttner, 2018^7^ | Switzerland, Poland, Israel | Hospitalized and ambulatory patients | 2013–2017 | 513 | Positive for nitrites or leukocyte esterase and urine culture ≥10^3^ cfu/mL | At least 1 out of 4 symptoms |  |
| Liu, 2019^8^ | China | One hospital and 2 community health centers (Wangjing) | 2010–2013 | 122 | Recurrent UTI: >10^5^ cfu/mL of a bacterial pathogen in the previous year | With symptoms |  |
| Matsumoto, 2011^9^ | Japan | 7 Medical and 3 urology clinics | 2008 | 39 | Not specified | Not specified |  |
| Monsen, 2014^10^ | Sweden | 18 primary healthcare centers | 1995–1998 | 1143 | - | Symptoms |  |
| Rădulescu, 2020^11^ | Romania | Hospital nephrology and dialysis department | 2018 | 93 | - | With symptoms |  |
| Sadahira, 2017^12^ | Japan | 12 Hospital or urology clinics | 2012–2014 | 104 | Pyuria (≥10 WBCs/mL or ≥10 WBCs/mm^3^ or positive leucocyte esterase or >5 WBCs/hpf centrifuged sediment) | Afebrile with symptoms |  |
| Wagenlehner, 2024^13^* | Multinational (mainly US, Europe) | 219 outpatients, general practice, obstetrics/gynecology, and urology clinics | 2019–2022 | 1201 | Pyuria (>15 WBC per hpf or leukocyte esterase 3+) | ≥2 symptoms |  |

cfu: colony-forming units, hpf: high-powered field, N: number of patients, US: United States, UTI: urinary tract infection, WBCs: white blood cells

*reports two clinical trials

**Table S2** Summary of key features of non-interventional observational studies reporting treatment failure

| **Reference** | **Country** | **Data source** | **Study years** | **N evaluated** | **UTI definition** | | | | |  |
| --- | --- | --- | --- | --- | --- | --- | --- | --- | --- | --- |
|  |  |  |  |  | **Microbiology** | **Symptoms** | **Antibiotic prescription** | **Disease codes** |  |  |
| **Database studies** | | | | | | | | | | |
| Butler, 2021^14^ | US | IBM MarketScan | 2006–2015 | 1,140,602 | - | - | On or after the diagnosis date (oral) | ICD-9-CM 595.0, 595.9, 599.0 codes |  |  |
| Franklin, 2023^15^ | US | Optum Clinformatics Data Mart Database | 2015–2021 | 238,335 | - |  |  | UTI code + antibiotic prescription with 1-year pre- and post-index follow-up |  |  |
| Fromer, 2023^16^ | US | Optum Clinformatics Data Mart Database | 2017–2022 | 376,004 | - |  | With 5 days of the diagnosis date | ICD-10-CM N30.0: acute cystitis, N30.9: cystitis unspecified, N39.0: UTI unspecified |  |  |
| Kon, 2022^17^ | US | Denver Health warehouse | 2018 | 623 | - | - | - | Three ICD-10 codes: Cystitis NOS (N39.0), Acute cystitis with (N30.01), and Acute cystitis without hematuria (N30.00). |  |  |
| Patel, 2023^18^  (Retrospective) | US | BD Insights Research Database | 2015-2022 | 2880 | Positive urine culture | - | Within 1 day of culture | - |  |  |
| Ten Doesschate, 2019^19^ | Netherlands | Julius General Practitioners’ Network | 2013–2018 | 46,855 | - | - | Antibiotic prescription | ICPC codes |  |  |
| Ten Doesschate, 2020^20^ | Netherlands | Julius General Practitioners' Network | 2013–2019 | 31,014 | - | - | Antibiotic prescription | ICPC codes (complicated and uncomplicated UTIs) reported |  |  |
| Shafrir, 2023^21^ | Israel | Meuhedet Health Services Database EHRs | 2013–2018 | 33,759 | - | - | Antibiotic prescription | ICD-9 diagnosis codes 599, 595.5, 595, 595.4, 595.8, 595.9, 595.89 |  |  |
| Schultze, 2023^22^ | Germany | WIG Benchmark database | 2013–2019 | 124,971 | - | - | Antibiotic prescription | ICD-10-CM N30.0: acute cystitis, N30.9: cystitis unspecified, N39.0: UTI unspecified |  |  |
| Wang, 2020^23^ | US | IBM MarketScan | 2013–2017 | 557,669 | - | - |  | Not specified |  |  |
| **Individual medical record review** | | | | | | | | | | |
| Ahn, 2021^24^  (Retrospective) | South Korea | Korea University Guro Hospital | 2016–2018 | 47 | Pyuria >5 WBCs/hpf; culture-proven ESBL-producing *Escherichia coli* or *Klebsiella pneumoniae* urine (≥10^5^ cfu/mL) | At least 1 of dysuria, frequency, urgency, or suprapubic pain | - | - |  |  |
| Beahm, 2018^25^  (Prospective) | Canada | Registry of 39 community pharmacies | 2017–2018 | 750 | - | Symptoms of UTI with no prescription | Presentation with prescription from another healthcare provider | - |  |  |
| Cowart, 2019^26^  (retrospective) | US | EHRs from 6 family medicine clinics | 2016 | 436 | - | - | Antibiotic prescription at the same visit | UTI as primary or secondary diagnosis code |  |  |
| Daumeyer, 2023^27*^  (Retrospective) | US | UTI telemedicine program | 2017–2021 | 2193 | - | UTI symptoms | - | - |  |  |
| Etani, 2017^28^  (Retrospective) | Japan | Komono Kosei Hospital urology department chart review | 2015–2017 | 155 | Pyuria (≥5 WBCs per hpf) and bacteriuria ≥10^3^ cfu/mL | Bladder irritation | - | - |  |  |
| Koguchi, 2020^29^  (Retrospective) | Japan | Hospital medical records | 2016–2019 | 223 | Bacterial count ≥10^4^ cfu/mL and pyuria (≥5 WBC per hpf) | Symptoms of acute UTI | - | - |  |  |
| Koh, 2023^30^  (Retrospective) | Singapore | 6 primary care clinics | 2019–2021 | 3194 | - | - | Antibiotic prescription | ICD-10 N28.9, N39.0 |  |  |
| Kusin, 2023^31**^  (prospective) | US | Urology clinic at a single institution | 2020–2022 | 65 | Symptoms of acute UTI | - | - | - |  |  |
| Naber, 2023^325^  (Retrospective) | Germany | General Practitioner chart review | 2017–2019 | 386 | Positive urine culture for *E. coli* | - | - | - |  |  |
| Yetsko, 2023^33^  (Retrospective) | US | EHRs rom outpatient settings across Trinity Health Michigan | 2020–2022 | 261 | Positive culture | Dysuria, suprapubic pain, frequency or urgency | Treated with cephalexin | ICD-10 N39.0 |  |  |
| Welch, 2021^34^  (Retrospective) | US | Michael E DeBakey Veterans Affairs Medical Center chart review | 2018–2019 | 278 | Leukocyte esterase positive, >2 RBC per hpf, >10 WBC per hpf, or positive for bacteria.  Patients with symptoms but no urinalysis result were included | UTI symptoms or altered mental health status with no alternative diagnosis | Antibiotic prescription | - |  |  |

cfu: colony-forming units, hpf: high-powered field, EHR: electronic healthcare records, ESBL: extended spectrum beta-lactamase, ICPC: International Classification of Primary Care, ICD-9/-10: International Classification of Disease 9^th^/10^th^ revision, ICD-9/-10-CM: International Classification of Disease 9^th^/10^th^ revision Clinical Modification, N: number of patients, US: United States, UTI: urinary tract infection, RBC: red blood cells, WBCs: white blood cells

*included patients with complicated and uncomplicated UTI

**74% of patients had recurrent UTI

**Table S3** Cochrane Risk of Bias Assessment for randomized controlled trials ^35^

| First author, year | Bias arising from the randomization process | Bias due to deviations from intended interventions | Bias due to missing outcome data | Bias in measurement of the outcome | Bias in selection of the reported result | Overall bias |
| --- | --- | --- | --- | --- | --- | --- |
| Dunne, 2023^3^ | Low risk | Low risk | Low risk | Some concerns | Low risk | Low risk |
| Hamasuna, 2014^4^ | Low risk | Low risk | Low risk | Low risk | Low risk | Low risk |
| Hassan, 2022^5^ | Some concerns | High risk | Low risk | High risk | Some concerns | High risk |
| Hooton, 2012^6^ | Low risk | Low risk | Low risk | Low risk | Low risk | Low risk |
| Huttner, 2018^7^ | Low risk | Some concerns | Low risk | Low risk | Low risk | Some concerns |
| Liu, 2019^8^ | Low risk | Low risk | Low risk | Low risk | Low risk | Low risk |
| Rădulescu, 2020^11^ | Low risk | Low risk | Low risk | Low risk | Low risk | Low risk |
| Sadahira, 2016^12^ | Low risk | Some concerns | Some concerns | Some concerns | Low risk | Some concerns |
| Monsen, 2014^10^ | Low risk | Low risk | Some concerns | Some concerns | Low risk | Some concerns |

**Table S4** Quality assessment of non-comparative observational studies^36^

| Questions | Naber, 2023^32^ | Abrahamian, 2011^1^ | Ahn, 2021^24^ | Choi, 2015^2^ |
| --- | --- | --- | --- | --- |
| 1. Were the two groups similar and recruited from the same population? | Y | Y | Y | UC |
| 2. Were the exposures measured similarly to assign people to both exposed and unexposed groups? | Y | Y | NA | NA |
| 3. Was the exposure measured in a valid and reliable way? | Y | Y | Y | UC |
| 4. Were confounding factors identified? | Y | N | N | N |
| 5. Were strategies to deal with confounding factors stated? | Y | N | N | N |
| 6. Were the groups/participants free of the outcome at the start of the study? | Y | Y | NA | N |
| 7. Were the outcomes measured in a valid and reliable way? | Y | Y | Y | Y |
| 8. Was the follow-up time reported and sufficient to be long enough for outcomes to occur? | Y | Y | Y | Y |
| 9. Was follow-up complete, and if not, were the reasons to loss to follow-up described and explored? | NA | N | Y | UC |
| 10. Were strategies to address incomplete follow up utilized? | NA | UC | NA | NA |
| 11. Was appropriate statistical analysis used? | Y | Y | N | Y |

Y, yes; N, no; UC, unclear; NA, not applicable**Table S5** Quality assessment of comparative observational studies^37^

| Bias Domain | Questions | Shafrir, 2023^21^ | Ten Doesschate, 2019^19^ | Yetsko, 2023^33^ | Beahm, 2018^25^ | Butler, 2021^14^ | Cowart, 2019^26^ | Daumeyer, 2023^27^ | Etani, 2017^28^ | Koguchi, 2020^29^ | Koh, 2023^30^ | Kon, 2022^17^ | Kusin, 2023^31^ | Matsumoto, 2011^9^ | Welch, 2021^34^ | Ten Doesschate, 2019^20^ |
| --- | --- | --- | --- | --- | --- | --- | --- | --- | --- | --- | --- | --- | --- | --- | --- | --- |
| **Selection** | 1. Representativeness of the exposed cohort | 1 | 1 | 1 | 1 | 1 | 1 | 1 | 1 | 1 | 1 | 1 | 1 | 1 | 1 | 1 |
|  | 2. Selection of non-exposed cohort | 1 | 1 | 1 | 1 | 1 | 1 | 1 | 1 | 0 | 1 | 1 | 1 | 0 | 0 | 1 |
|  | 3. Ascertainment of exposure | 1 | 1 | 1 | 1 | 1 | 1 | 0 | 1 | 1 | 1 | 1 | 1 | 1 | 1 | 1 |
|  | 4. Demonstration that outcome of interest was not present at start of study | 1 | 1 | 1 | 1 | 1 | 1 | 1 | 1 | 1 | 1 | 1 | 1 | 1 | 1 | 1 |
| **Comparability** | Comparability of cohorts on the basis of the design or analysis | 2 | 2 | 2 | 0 | 0 | 1 | 0 | 0 | 0 | 1 | 0 | 1 | 0 | 1 | 1 |
| **Outcome** | 1. Assessment of outcome | 1 | 1 | 1 | 0 | 1 | 1 | 0 | 1 | 1 | 1 | 1 | 1 | 1 | 1 | 1 |
|  | 2. Was follow-up long enough for outcomes to occur | 1 | 1 | 1 | 1 | 1 | 1 | 1 | 0 | 1 | 1 | 1 | 1 | 1 | 1 | 1 |
|  | 3. Adequacy of follow-up of cohorts | 1 | 1 | 1 | 1 | 1 | 1 | 0 | 0 | 1 | 1 | 1 | 1 | 1 | 1 | 1 |

A study can be awarded a maximum of one star for each numbered item within the Selection and Exposure categories. A maximum of two stars can be given for Comparability.

**Table S6** Summary of antibiotic interventions and treatment resistance at enrollment (or the earliest available culture) in clinical trials and studies using individual patient data extracted from health records included in this review

| **Author, years** | **Intervention** | **Dose and schedule** | **% resistant to the prescribed antibiotic** |
| --- | --- | --- | --- |
| Abrahamian, 2011^1^ | TMP/SMX | 160/800 mg BID for 3 days | 25.2% |
| Choi, 2015^2^ | Ciprofloxacin | 250 mg BID for 5 days | Not reported |
| Dunne, 2023^3^ | Sulopenem etzadroxil, | 500 mg/500 mg BID for 5 days | 29.0% ciprofloxacin non-susceptible |
|  | Ciprofloxacin | 250 mg BID for 3 days | 25.8% |
| Hamasuna, 2014^4^ | Faropenem | 200 mg TID for 3 days | 9.2% fluoroquinolone resistant |
|  | Faropenem | 200 mg TID for 7 days | 3.4% ESBL-producing |
| Hassan, 2022^5^ | Fosfomycin | 3 g (single dose) | Not reported |
|  | Ciprofloxacin | 500 mg daily for 5 days | Not reported |
| Hooton, 2012^6^ | Ciprofloxacin | 250 mg BID for 3 days | 4% |
|  | Cefpodoxime | 100 mg BID for 3 days | 8% |
| Huttner, 2018^7^ | Nitrofurantoin | 100 mg TID for 5 days | *E. coli* 1%, *Klebsiella* spp 15%, *Proteus* spp 86%, *Enterococcus* spp 7%, *Enterobacter* spp 3%, Group B *Streptococcus* 4%, Mixed flora 26%, Other 5% |
|  | Fosfomycin | 3 g (single dose) | *E. coli* 1%, *Klebsiella* spp 0%, *Proteus* spp 20%, *Enterococcus* spp 7%, *Enterobacter* spp 2%, Group B *Streptococcus* 3%, Mixed flora 21%, Other 4% |
| Liu, 2019^8^ | Levofloxacin | 200 mg BID for 1 week | 70.5% resistant to 1 antibiotic, 40.7% resistant to 5 antibiotics |
|  | Amoxicillin/clavulanic acid | 500 mg TID for 1 week |  |
| Matsumoto, 2011^9^ | Fosfomycin calcium | 1 g TID for 2 days | 0% |
| Monsen, 2014^10^ | Pivmecillinam | 200 mg TID for 7 days  200 mg BID for 7 days 400 mg BID for 3 days | 16.4% |
|  | Placebo | - |  |
| Rădulescu, 2020^11^ | TMP-SMX | For 7 days | 53.3% |
| Sadahira, 2017^12^ | Cefditoren pivoxil | 100 mg TID for 3 days | 11.9% fluoroquinolone-non-susceptible  8.3% were ESBL-producing *E. coli* |
|  | Cefditoren pivoxil | 100 mg TID for 7 days |  |
| Wagenlehner, 2024^13^ | Gepotidacin | 1500 mg BID for 5 days | 51.1%/52.9% *E. coli* multi-drug resistant |
|  | Nitrofurantoin | 100 mg BID for 5 days | 44.3%/38.6% *E. coli* multi-drug resistant |
| Ahn, 2021^24^ | Amikacin and amoxicillin/clavulanate | 250 mg intramuscular amikacin (single dose), amoxicillin/clavulanate 500/125 mg for 7 days | All ESBL-producing *E. coli* or *K. pneumoniae*  0% resistance to amikacin, 8.5% resistant to amoxicillin/clavulanate |
| Beahm, 2018^25^ | Numerous | Not reported | Not reported |
| Cowart, 2019^26^ | Numerous | Not reported | Not reported |
| Daumeyer, 2023^27^ | Numerous | Not reported | Not reported |
| Etani, 2017^28^ | Cefaclor | 750 mg daily for 7 days | 13.0% (*E.coli*) |
| Koguchi, 2020^29^ | Cefaclor | 750 mg daily for 5–7 days | 15.9% |
| Koh, 2023^30^ | TMP-SMX | Not reported | 17.4% (*E. coli*) |
|  | Amoxicillin/clavulanate | - | 7.4% (*E. coli*) |
|  | Nitrofurantoin | - | 0.6% (*E. coli*) |
|  | Ciprofloxacin | - | 20.0% (*E. coli*) |
| Kusin, 2023^31^ | Nitrofurantoin | 100 mg BID for 7 days | 11% |
| Naber, 2023^32^ | Fosfomycin-trometamol | 3 g (single dose) | 29.0% resistant to one or two drug classes  3.9% multi-drug resistant |
|  | Nitrofurantoin | 200–300 mg daily for 3–7 days |  |
| Yetsko, 2023^33^ | Cephalexin | 500 mg BID for 5–7 days | Patients with resistant organisms excluded |
|  | Cephalexin | 500 mg QID for 5–7 days |  |
| Welch, 2021^34^ | Nitrofurantoin | 100 mg BID | Patients with resistant organisms excluded |

BID: twice daily, ESBL: extended spectrum beta-lactamases, QID: four times daily, TID: three times daily, TMP-SMX: trimethoprim-sulfamethoxazole

**REFERENCES**

1. Abrahamian F, Krishnadasan A, Mower W et al. The association of antimicrobial resistance with cure and quality of life among women with acute uncomplicated cystitis. Infection 2011; 39: 507-14.

2. Choi H, Kim Y, Bae J. Quality of life and changes in symptom relief in patients with acute uncomplicated cystitis treated with antibiotics: a prospective, open-label, multicenter, observational study. Eur J Clin Microbiol Infect Dis 2015; 34: 1119-24.

3. Dunne MW, Aronin SI, Das AF et al. Sulopenem or ciprofloxacin for the treatment of uncomplicated urinary tract infections in women: a phase 3, randomized trial. Clin Infect Dis 2023; 76: 66-77.

4. Hamasuna R, Tanaka K, Hayami H et al. Treatment of acute uncomplicated cystitis with faropenem for 3 days versus 7 days: multicentre, randomized, open-label, controlled trial. J Antimicrob Chemother 2014; 69: 1675-80.

5. Hassan MM, Malik M, Saleem R et al. Efficacy of single dose of fosfomycin versus a five-day course of ciprofloxacin in patients with uncomplicated urinary tract infection. Cureus 2022; 14: e24843

6. Hooton TM, Roberts PL, Stapleton AE. Cefpodoxime vs ciprofloxacin for short-course treatment of acute uncomplicated cystitis: a randomized trial. JAMA 2012; 307: 583-9.

7. Huttner A, Kowalczyk A, Turjeman A et al. Effect of 5-day nitrofurantoin vs single-dose fosfomycin on clinical resolution of uncomplicated lower urinary tract infection in women: a randomized clinical trial. JAMA 2018; 319: 1781-9.

8. Liu S-w, Guo J, Wu W-k et al. Treatment of uncomplicated recurrent urinary tract infection with Chinese medicine formula: a randomized controlled trial. Chin J Integrative Med 2019; 25: 16-22.

9. Matsumoto T, Muratani T, Nakahama C et al. Clinical effects of 2 days of treatment by fosfomycin calcium for acute uncomplicated cystitis in women. J Infect Chemother 2011; 17: 80-6.

10. Monsen TJ, Holm SE, Ferry BM et al. Mecillinam resistance and outcome of pivmecillinam treatment in uncomplicated lower urinary tract infection in women. Apmis 2014; 122: 317-23.

11. Rădulescu D, David C, Turcu FL et al. Combination of cranberry extract and D-mannose-possible enhancer of uropathogen sensitivity to antibiotics in acute therapy of urinary tract infections: Results of a pilot study. Exp Ther Med 2020; 20: 3399-406.

12. Sadahira T, Wada K, Araki M et al. Efficacy and safety of 3 day versus 7 day cefditoren pivoxil regimens for acute uncomplicated cystitis: multicentre, randomized, open-label trial. J Antimicrob Chemother 2017; 72: 529-34.

13. Wagenlehner F, Perry CR, Hooton TM et al. Oral gepotidacin versus nitrofurantoin in patients with uncomplicated urinary tract infection (EAGLE-2 and EAGLE-3): two randomised, controlled, double-blind, double-dummy, phase 3, non-inferiority trials. Lancet 2024; 403: 741-55.

14. Butler AM, Durkin MJ, Keller MR et al. Risk of antibiotic treatment failure in premenopausal women with uncomplicated urinary tract infection. Pharmacoepidemiol Drug Saf 2021; 30: 1360-70.

15. Franklin M, Emden MR, Kautz S et al. 2839. Cost burden of patients with oral antibiotic treatment failure for uncomplicated urinary tract infection in the United States. Open Forum Infect Dis 2023; 10: ofad500.2449.

16. Fromer DL, Luck ME, Cheng WY et al. 2828. Incidence of treatment failure when treated with empiric oral antibiotics among us female outpatients with uncomplicated urinary tract infection. Open Forum Infect Dis 2023; 10: ofad500.2439.

17. Kon S, Meslovich D, Valdez C et al. Long-term impact of fluoroquinolone-sparing strategies for empirical treatment of acute uncomplicated cystitis among ambulatory patients. Ther Adv Infect Dis 2022; 9: 20499361221129415.

18. Patel R GV, Preib M, Ye G, Joshi A, Mitrani-Gold F, Mulgirigama A, Yu K, Kaye K. Impact of extended-spectrum ß-lactamase-positivity in urine isolates on outcomes in female patients with uncomplicated urinary tract infection. P1376. 33rd European Congress of Clinical Microbiology & Infectious Diseases. 15-8 April, 2023. Copenhagen, Denmark. 2023.

19. Ten Doesschate T, Groenwold RH, Bonten MJ et al. Effectiveness of extended-versus normal-release nitrofurantoin for cystitis: an instrumental variable analysis. J Antimicrob Chemother 2019; 74: 3337-43.

20. Ten Doesschate T, van Haren E, Wijma RA et al. The effectiveness of nitrofurantoin, fosfomycin and trimethoprim for the treatment of cystitis in relation to renal function. Clin Microbiol Infect 2020; 26: 1355-60.

21. Shafrir A, Oster Y, Shauly-Aharonov M et al. Real-life comparison of fosfomycin to nitrofurantoin for the treatment of uncomplicated lower urinary tract infection in women. Biomedicines 2023; 11: 1019.

22. Schultze M MA, Pignot M, Krinner A, Kossack N, Mitrani-Gold FS, Joshi A. Recurrence, healthcare resource use, and costs in the treatment of uncomplicated urinary tract infection in female patients in Germany. E0351. 33rd European Congress of Clinical Microbiology & Infectious Diseases. 15-8 April, 2023. Copenhagen, Denmark. 2023.

23. Wang Y, Mitrani-Gold FS, Xie L et al. 123. Treatment patterns and prevalence of inappropriate and suboptimal antibiotic use among females with uncomplicated urinary tract infection in the US. Open Forum Infect Dis 2020; 7: S190-S1.

24. Ahn ST, Lee DH, Kim JW et al. Single-dose amikacin plus 7 days of amoxicillin/clavulanate to treat acute cystitis caused by extended-spectrum beta-lactamase-producing Escherichia coli: A retrospective cohort study. Investig Clin Urol 2021; 62: 310-6.

25. Beahm NP, Smyth DJ, Tsuyuki RT. Outcomes of urinary tract infection management by pharmacists (RxOUTMAP): a study of pharmacist prescribing and care in patients with uncomplicated urinary tract infections in the community. Can Pharm J (Ott) 2018; 151: 305-14.

26. Cowart K, Worley M, Rouby NE et al. Evaluation of FDA boxed warning on prescribing patterns of fluoroquinolones for uncomplicated urinary tract infections. Ann Pharmacother 2019; 53: 1192-9.

27. Daumeyer NM, Kreitzberg D, Gavin KM et al. Real-world evidence: Telemedicine for complicated cases of urinary tract infection. PLoS One 2023; 18: e0280386.

28. Etani T, Naiki T, Yamaguchi S et al. Antimicrobial susceptibility of pathogens in acute uncomplicated cystitis cases in the urology department of a community hospital in Japan: comparison with treatment outcome and hospital-wide antibiogram. J Infect Chemother 2017; 23: 692-7.

29. Koguchi D, Murakami Y, Ikeda M et al. Cefaclor as a first-line treatment for acute uncomplicated cystitis: a retrospective single-center study. BMC Urol 2020; 20: 1-7.

30. Koh SWC, Ng TSM, Loh VWK et al. Antibiotic treatment failure of uncomplicated urinary tract infections in primary care. Antimicrob Resist Infect Control 2023; 12: 73.

31. Kusin SB, Fan EM, Prokesch BC et al. Empiric versus culture-based antibiotic therapy for UTIs in menopausal women. World J Urol 2023; 41: 791-6.

32. Naber KG, Wagenlehner F, Kresken M et al. Escherichia coli resistance, treatment patterns and clinical outcomes among females with uUTI in Germany: a retrospective physician-based chart review study. Sci Rep 2023; 13: 12077.

33. Yetsko A, Draper HM, Eid K et al. Two times versus four times daily cephalexin dosing for the treatment of uncomplicated urinary tract infections in females. Open Forum Infect Dis 2023; 10: ofad430.

34. Welch E, Sheth S, Ashong CN et al. Retrospective review on the safety and efficacy of nitrofurantoin for the treatment of cystitis in the veteran population with or without renal insufficiency. Open Forum Infect Dis 2021; 8: ofab442.

35. Cochrane Methods Bias. RoB 2: A revised Cochrane risk-of-bias tool for randomized trials. <https://methods.cochrane.org/bias/resources/rob-2-revised-cochrane-risk-bias-tool-randomized-trials>.

36. JBI Critical Appraisal Tools. <https://jbi.global/critical-appraisal-tools>

37. Wells G, Shea B, O'Connell D et al. The Newcastle-Ottawa Scale (NOS) for assessing the quality of nonrandomised studies in meta-analyses, 2021. <https://www.ohri.ca/programs/clinical_epidemiology/oxford.asp>.
